# Are you willing to forgive generative AI doctors? Trust repair after failures in online health consultation services

**DOI:** 10.3389/fpsyg.2025.1668633

**Published:** 2025-10-24

**Authors:** Yanjie Chen, Shujun Luo, Yukun Yin

**Affiliations:** ^1^Faculty of Humanities and Arts, Macau University of Science and Technology, Macau, China; ^2^Media Art Research Center, Jiangxi Institute of Fashion Technology, Nanchang, China; ^3^School of Sociology and Humanities, Jiangxi University of Finance and Economics, Nanchang, China

**Keywords:** generative artificial intelligence (GAI), attribution style, social support, anthropomorphism, trust repair, online health consultation services (OHCSV)

## Abstract

While generative AI doctors are increasingly used in online health consultation services, research on trust repair following service failures remains limited. We examined how attribution style, social support, and anthropomorphism influence individuals’ trust repair and behavioral intention. A total of 512 participants were recruited to take part in a between-subjects experiment with a 2 (internal vs. external attribution) × 2 (informational vs. emotional support) × 2 (anthropomorphism vs. non-anthropomorphism) design. The results revealed that participants exposed to internal attribution, emotional support, or anthropomorphism conditions reported higher levels of trust repair. Anthropomorphism influences the effectiveness of attribution style and social support in repairing trust in GAI doctors. Moreover, an interesting interaction was observed between attribution style and social support: when the GAI doctor used internal attribution, informational support was more effective; under external attribution, emotional support proved more effective. In addition, the effect of social support on behavioral intention was fully mediated by trust repair. These findings offer practical implications for optimizing the design of GAI doctors, enhancing communication and collaboration between GAI doctors and users, and ultimately strengthening the resilience of AI-based health consultation services.

## Introduction

In recent years, generative artificial intelligence doctors (GAI doctors) have emerged as a new form of medical assistance and are being widely adopted in online health consultation services (OHCSV; Guo and Chen, 2025; Li, Y et al., 2025). Powered by advanced algorithms, GAI doctors are capable of producing predetermined responses through the analysis of user inputs and retrieval of relevant medical knowledge (Chow et al., 2024). Therefore, compared with human doctors, GAI doctors can provide round-the-clock services, overcome geographical limitations, and supplement scarce medical resources. However, realization of GAI doctors’ potential relies heavily on user trust, and low trust or any breach of trust may undermine users’ continued engagement with these systems (Li and Liu, 2025; Li, Y et al., 2025). Consequently, many previous studies have focused on how to establish and enhance individuals’ trust in GAI doctors (Chen and Cui, 2025; Detjen et al., 2025; Kim et al., 2024). Nevertheless, these studies have mainly addressed the development of general trust, paying little attention to trust repair following service failures. Like any other AI service, GAI doctors are not perfect (Chen et al., 2022). They might also fail, such as providing inaccurate diagnoses, failing to detect important symptoms, or providing suboptimal recommendations with insufficient information. However, unlike other general AI service failures, failures in GAI doctors might cause significant health issues so that people use GAI doctors to seek health care with caution and scrutiny (Quinn et al., 2021). That is, service failures by GAI doctors may notably weaken users’ trust and reduce their intention to keep using such services. Hence, focusing on trust repair following service failures of GAI doctors is both practically and theoretically important.

Existing research in the field of human–machine interaction (HMI) indicates that the way trustees attribute the causes of failures significantly influences the trustor’s perception of the event (Chen et al., 2022; Kim and Song, 2021). Providing social support by GAI doctors helps enhance individuals’ positive expectations toward them (Li et al., 2025; Zhou and Chang, 2024). Endowing GAI doctors with human-like characteristics can improve the resilience of users’ trust (De Visser et al., 2016; Li et al., 2023). Despite considerable research on attribution style and anthropomorphism in trust repair, little is known about how these factors affect trust restoration in health consultation scenarios involving GAI doctors. Different forms of social support have been found to affect trust in GAI doctors, but they have seldom been studied in the context of repairing trust after failures. The advancement of medical AI should emphasize human-centered design and trustworthiness (Albahri et al., 2023). In line with this, the present study primarily examines how attribution style, social support, and anthropomorphism influence trust repair in the context of medical AI service failures. In addition, we investigate how trust repair shapes the relationship between social support and behavioral intentions. Gaining insight into these processes can enhance the adaptability and resilience of GAI-based health consultation systems.

### Trust and trust repair

In the context of HMI, trust can be defined as the belief or attitude that an agent will assist in achieving an individual’s goals in situations characterized by uncertainty and vulnerability (De Visser et al., 2016). Although many scholars define trust and use it as a baseline to study repair, general trust and trust repair differ both qualitatively and quantitatively. From a qualitative perspective, general trust develops under the assumption of “trustworthy until proven otherwise,” whereas trust repair occurs after this assumption is violated, with betrayal not only damaging prior trust but also triggering negative emotions and concerns about further harm (Kim et al., 2004; Sharma et al., 2023). Thus, while the essence of general trust lies in fostering positive expectations, trust repair additionally requires addressing post-violation negative effects to restore the relationship. From a quantitative perspective, in the initial stage of a relationship, individuals often exhibit relatively high levels of trust based on cues such as trust propensity, sense of dependence, institutional safeguards, and group identity or reputation (Kim et al., 2004, 2009). However, once a violation occurs, trust can easily fall below its initial level, and the magnitude of increase required to rebuild trust is substantially greater than that needed to establish initial trust (Kim et al., 2004, 2006; Lewicki and Brinsfield, 2017). In summary, trust repair is more complex and challenging than the initial development of general trust. Therefore, this study adopts the definition by Sharma et al. (2023), which states that “trust repair was any increase in trust above the post-transgression level and complete repair as an increase in trust to the pre-transgression level.” This definition not only captures the dynamic changes in trust following a violation but also provides a clear operational standard for empirical analysis.

**Figure 1:**
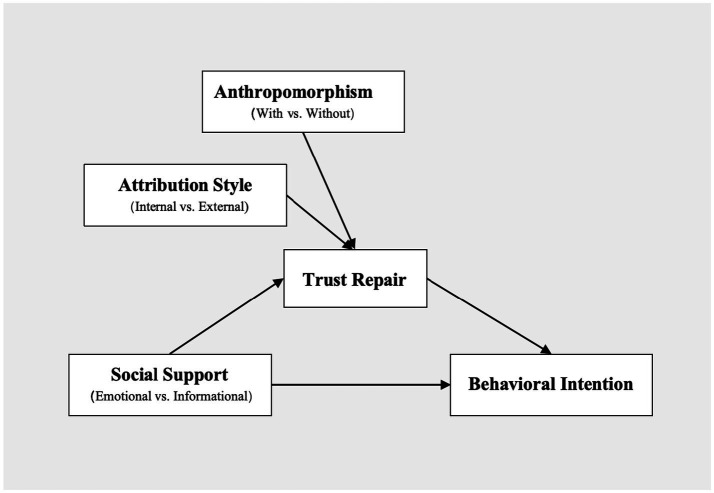
Conceptual model in the current study.

For many years, researchers have focused on exploring the factors and mechanisms that affect trust repair. In general, mechanisms for trust repair can be categorized into attribution, social-equilibrium, and structural mechanisms (Dirks et al., 2009; Sharma et al., 2023). According to attribution mechanisms, after a trust violation occurs, how the trustor attributes the failure plays a major role in restoring the relationship with the trustee (Kim et al., 2009; Tomlinson and Mayer, 2009). Social equilibrium mechanisms suggest that a trust violation disrupts the trust established between parties based on existing social norms, requiring restorative measures, particularly those aimed at alleviating negative emotions, to repair the relationship (Gillespie and Siebert, 2018; Ren and Gray, 2009). Structural mechanisms posit that if the external environment facilitates trust or reduces the likelihood of untrustworthy behaviors, trust can be more effectively restored (Dirks et al., 2009; Sitkin & Roth, 1993). Overall, trust repair primarily involves three dimensions: attribution of the breach, the relationship, and the environment (Sharma et al., 2023). Trust is more likely to be repaired if individuals perceive the attribution of responsibility as acceptable, the damaged relationship is mended, and the environment supports trust. Therefore, based on these three mechanisms, this study aims to examine how attribution style, social support, and anthropomorphism influence trust repair and behavioral intentions in GAI doctors (see Figure 1).

### Attribution theory and trust repair

According to attribution theory, attribution constitutes a fundamental cognitive process (Chen et al., 2022; Weiner, 1985). Through this process, individuals seek to identify the causes of behavioral events in order to enhance their understanding of the internal and external world. In general, attributions can be divided into internal and external types. In the context of service failures in HMI, it typically represents different ways of taking responsibility. Specifically, internal attribution means that the GAI takes active responsibility for a service failure, such as attributing it to the use of inaccurate data (Kim and Song, 2021). Conversely, external attribution occurs when the GAI places the cause of a service failure on external factors, such as environmental conditions or human interference (Zhang et al., 2023). Based on expectation confirmation theory, when the attribution style used by a GAI matches individuals’ expectations, it is more likely to satisfy their psychological needs and facilitate trust repair (Oliver, 1980). If the attribution style does not match expectations, it could make the negative effects even worse. Studies have shown that following a trust violation, a machine taking responsibility proactively helps repair trust because it signals sincere regret (Kim et al., 2006; Ohbuchi et al., 1989; Tomlinson et al., 2004). However, some studies suggest that proactively taking responsibility does not always produce positive outcomes. For example, Kim and Song (2021) found that when an anthropomorphized AI issued an apology based on external rather than internal attribution, it resulted in greater trust repair. Furthermore, some researchers have found that internal attribution tends to elicit blame from the victim, whereas external attribution does not, as people recognize that many events are influenced by external factors (Kim et al., 2006; Sullivan, 1975). Yet, external attributions are not without drawbacks. When trustors question the agent’s innocence, such attributions may be perceived as excuses or indications of incompetence (Schlenker et al., 2001). Kim et al. (2006) found that in human-to-human interaction (HHI), internal attributions for competence-related failures are more effective than external attributions in repairing trust, as they convey responsibility and integrity to the trustor and, more importantly, signal a greater likelihood of correcting the behavior in the future. GAI, supported by large-scale machine learning models, can continuously optimize its algorithms through iterative training, thereby enhancing the quality and adaptability of its outputs (Qin et al., 2025). Therefore, in the context of this study, we propose the following hypothesis:

*H*1: Compared with external attributions, internal attributions will result in higher trust repair.

### Social support and trust repair

Prior studies have shown that trust violations in HMI may be alleviated through trust repair strategies, such as the provision of recovery services (Kim and Song, 2021; Meng et al., 2025). More specifically, service recovery involves the actions a provider implements following a service failure, aimed at mitigating customer dissatisfaction and resolving complaints—typically through apology, compensation, and restoration (Spreng et al., 1995; Zhou and Chang, 2024). According to social support theory, individuals’ access to supportive relationships or resources—primarily in the form of informational support and emotional support—can have a positive impact on their well-being (Langford et al., 1997). Informational support means offering useful guidance and advice to assist individuals in solving problems and making informed decisions (Madjar, 2008). Emotional support involves the expression of love, empathy, and understanding, allowing individuals to feel cared for and understood (Reblin and Uchino, 2008). Accordingly, social support theory has been extensively used in trust-building research. However, few studies have examined how social support influences individuals’ trust repair, particularly in the context of AI-based health consultations. Specifically, in the domain of OHCSV, GAI doctors can provide informational service recovery by explaining the reasons for service failures and offering additional informational support to help individuals address their concerns (Zhou and Chang, 2024). Previous research indicates that due to the black-box nature of AI, lay users often lack understanding of how decisions or results are generated. Therefore, informing users about the AI system’s data processing and operational mechanisms is considered an effective approach to enhancing user trust (Afroogh et al., 2024; Felzmann et al., 2019). In other words, a substantial body of prior research has demonstrated that the provision of transparent information helps users feel neither deceived nor compelled. However, numerous studies have also demonstrated that trust is not a simple function of transparency; human-like features of robots, particularly emotional attributes, play a significant role in facilitating interaction between humans and AI (Gebhard et al., 2021; Troshani et al., 2021). Emotional service recovery can allow individuals to feel understood, empathized with, and comforted by the AI, thereby potentially alleviating the negative experiences caused by service failures. Given that, in the context of service failures during health consultations, individuals primarily experience pressure to obtain clear, accurate, and useful medical information to reduce uncertainty and guide their health decisions (Li, Y et al., 2025; Liu et al., 2022), we predict that informational support, compared with emotional support, will be more effective in facilitating trust repair.

*H*2: Compared with emotional support, informational support will result in higher trust repair.

Moreover, previous research has consistently shown that social support positively affects users’ behavioral intentions (Bu et al., 2024; Rashidi et al., 2025; Zhou and Chang, 2024); yet, service failures may weaken this effect, reducing continued engagement with GAI healthcare services. Trust is crucial in designing interactive intelligent agents, as it influences how individuals perceive, interact with, and evaluate technology (Kim and Song, 2021; Li et al., 2008). Based on this, we argue that in the context of GAI doctor service failures, trust repair may play a key role in the relationship between social support and behavioral intention. Accordingly, the following hypotheses are proposed:

*H*3a: Social support positively influences behavioral intention.

*H*3b: Trust repair mediates the relationship between social support and behavioral intention.

### Anthropomorphism and trust repair

With the rapid advancement of technologies such as robotics, automation, and natural language processing, the boundary between humans and machines has become increasingly blurred (De Visser et al., 2016). Robots are not only becoming more intelligent and capable of assisting humans across various domains, but are also increasingly anthropomorphized, as designers often incorporate human-like visual features, identity cues, or language to enhance their social presence (Go and Sundar, 2019). According to the Computers Are Social Actors (CASA) paradigm, enhancing the level of anthropomorphism in machines facilitates HMI by making the agent appear more familiar and trustworthy (Nass et al., 1994). In service recovery contexts, existing research similarly suggests that anthropomorphism improves consumer experience and enhances the effectiveness of service recovery. For example, Agnihotri and Bhattacharya (2024) demonstrated that anthropomorphism enhances consumers’ perceptions of a chatbot’s honesty and integrity, thereby increasing their willingness to forgive it for service failures. Zhou and Chang (2024) reported a positive association between higher levels of anthropomorphism and both perceived service quality and attitude satisfaction in service recovery contexts. Moreover, De Visser et al. (2016) found that anthropomorphism enhances trust resilience in cognitive agents. Although anthropomorphism’s positive effects on service recovery have been widely studied, its role in trust repair specifically within AI healthcare consultations receives limited attention. Li, Y et al. (2025) showed that in AI healthcare consultations, anthropomorphism boosts perceptions of a robot’s social presence, increasing source credibility and behavioral intentions. This suggests people apply different “humanness” heuristics when interacting with robots versus real humans, resulting in distinct psychological responses (Li, Y et al., 2025; Sundar, 2008). Based on this, the current study assumes that anthropomorphism also improves the effectiveness of trust repair in AI healthcare consultations. Accordingly, we propose the following research hypothesis:

*H*4: Compared with non-anthropomorphic GAI doctors, anthropomorphic GAI doctors will result in higher trust repair.

In addition to examining the main effects of attribution style, social support, and anthropomorphism on trust repair, this study also explores whether there are interaction effects among these factors. According to Kim and Song (2021), the lowest level of trust damage occurred when a machine-like agent used external rather than internal attributions. Li, Y et al. (2025) reported that anthropomorphic GAI doctors providing informational support can enhance their social presence, thereby increasing source credibility. Moreover, Chen et al. (2022) found that in cases of service failure with external attribution, recovery actions taken by the healthcare provider, rather than the consumer, were effective in restoring cognitive trust. Therefore, we hypothesize that attribution style, social support, and anthropomorphism interactively affect trust repair in GAI doctors:

*H*5: There is an interaction effect between attribution style, social support, and anthropomorphism on trust repair.

## Methods

### Participants

This study recruited 512 eligible participants through Credamo, an online experimental survey platform specializing in social science research in China. All participants were over 18 years old and met the inclusion criteria (see Table 1). They were randomly selected from Credamo’s managed respondent pool. We performed *a priori* power analysis with G*Power 3.1 software to confirm sufficient statistical power. The results presented that at least 210 participants were needed (power = 0.95, *α* = 0.05, effect size = 0.25), a requirement that our sample successfully fulfilled.

**Table 1:** Demographic characteristics of participants.

Demographics variable	Category	Frequency
Gender	Female	356
	Male	156
Age	< 20	4
	20–29	281
	30–39	189
	40–49	23
	50–59	14
	60+	1
Education	junior college or below	42
	Undergraduate	375
	Master’s degree and above	95
Frequency of using GAI doctors	< 5 times	1
	5–10 times	121
	11–15 times	263
	16–20 times	109
	> 20 times	18

### Design

Upon the approval of IRB of the author’s affiliated university (MUST-FA-20250017), we conducted an online experiment with a 2 (internal attribution vs. external attribution) × 2 (informational support vs. emotional support) × 2 (anthropomorphism vs. non-anthropomorphism) between-subjects factorial design. Two medical professionals were invited to review the AI-generated content for accuracy.

The experiment included two scenarios and three stages of trust measurement: initial trust, trust violation, and trust repair. Scenario 1 (Trust Violation) presented a text-only dialog in which the GAI doctor’s advice conflicted with participants’ prior knowledge, aiming to induce a decline in trust. Scenario 2 (Trust Repair) built upon Scenario 1, presenting the full dialog including the trust violation and the assigned recovery strategy, in order to examine how different combinations of attribution style, social support, and anthropomorphism influenced trust repair (see Supplementary materials). Notably, Scenario 1 constituted the first part of Scenario 2, since trust repair logically requires a prior violation. To prevent the manipulation of anthropomorphism from influencing the trust violation scenario, Scenario 1 was presented in a text-only format.

At the beginning of the experiment, participants reported their initial trust in the GAI doctor after providing informed consent, serving as a baseline measurement. Next, participants entered Scenario 1, where they were asked to imagine consulting the GAI doctor about fish oil consumption (viewing the stimulus for at least 15 s) and then report their trust in the GAI doctor. Subsequently, participants were randomly assigned to one of the eight experimental conditions (Scenario 2). During this scenario, participants viewed the full dialog between the GAI doctor and the patient (for at least 35 s) and then reported their trust in the doctor again. Additionally, participants reported their behavioral intentions and demographic information, including gender, age, education, and frequency of using GAI doctors. Finally, participants were explicitly informed that the information provided was fictitious and did not constitute real medical advice.

### Stimulus

For this study, the experimental dialog was set within a scenario in which users inquired about the appropriate dosage of fish oil supplements. This scenario was chosen due to the growing attention individuals pay to personal health management. Although people frequently purchase dietary supplements independently, they often lack sufficient knowledge regarding their necessity and correct usage. Within this health-consumption context, consulting GAI doctors has become a convenient way for individuals to access health advice.

Following previous research (Kim and Song, 2021), we manipulated attribution style by defining internal attribution as errors in AI health consultations caused by the system retrieving inaccurate information, and external attribution as errors resulting from insufficient information provided by the user. Accordingly, participants in the internal attribution condition were presented with a GAI doctor attributing the error to the AI system itself, whereas those in the external attribution condition saw the GAI doctor attributing the error to the user.

For social support, participants in the informational support condition were exposed to a GAI doctor that appeared objective and calm, offering detailed advice on fish oil supplementation. Example expressions included specific dosage recommendations such as, “Relevant studies suggest that a daily intake of 1,000 to 3,000 mg of fish oil is generally safe and beneficial for healthy adults,” along with links to additional web resources for further information. In the emotional support condition, participants were exposed to a GAI doctor conveying warmth and understanding. Example expressions included, “Dear friend, I truly understand your concern about your health, and I know how confusing it could be when faced with so much conflicting information. I’ll always be here with you, supporting and protecting your health.”

Moreover, we adopted the approach of manipulating anthropomorphic visual cues based on prior research (Go and Sundar, 2019; Li, Y et al., 2025). For participants in the anthropomorphism condition, the interaction interface featured a fictional GAI doctor with human-like characteristics. In contrast, those in the non-anthropomorphism condition viewed a standard ChatGPT dialog window.

### Measures

#### Trust repair

A three-item scale adapted from Meng et al. (2025) was used to measure trust repair, with participants rating each item on a 7-point Likert scale (1 = strongly disagree, 7 = strongly agree). The items were: (1) The GAI doctor gives me the impression of being trustworthy; (2) I consider the GAI doctor to be competent and reliable; (3) I think GAI doctors are willing to look after the health interests of patients (*M* = 3.876, *SD* = 1.854, *Cronbach’s α* = 0.894). Trust at the initial, trust violation, and trust repair stages was measured using the same scale.

#### Behavioral intention

A four-item scale adapted from Hadi et al. (2024) was used to measure behavioral intention, with participants rating each item on a 7-point Likert scale (1 = strongly disagree, 7 = strongly agree). The items were: (1) I intend to continue using AI health consultation; (2) Compared to other consultation methods, I am still willing to consult a GAI doctor; (3) I am willing to consult a GAI doctor again when I face health issues in the future; (4) It is unlikely that I will stop using AI health consultation because of a service failure problem (*M* = 4.254, *SD* = 2.499, *Cronbach’s α* = 0.941).

To assess the effectiveness of our experimental manipulations, we included three sets of manipulation check items in the questionnaire. For attribution, participants were invited to answer the question: “Was the service failure caused by the AI system retrieving inaccurate information?” To evaluate social support, participants rated the GAI doctor on perceived sympathy, inspiration, warmth, and care. Higher scores indicated a greater level of emotional support. For anthropomorphism, participants answered the question: “How do you think about the GAI doctor’s anthropomorphism capability?” A 7-point Likert scale (1 = strongly disagree, 7 = strongly agree) was used to assess all items.

## Results

### Data analysis

Since this study involved two scenarios and three stages of trust measurement, a paired-samples t-test was conducted to examine changes in trust across the stages. The results, presented in Table 2, indicate that trust significantly decreased following the service failure and was subsequently restored after recovery, regardless of the recovery method. These findings confirm that the manipulation was successful, allowing us to proceed with further analyses.

**Table 2:** The comparison among the trust in three stages.

Outcome variable	Stage	*M*	*SD*	*t-*value
Trust	Initial-violation	2.159	1.222	39.986^***^
	Violation-repaired	−0.687	1.300	−11.958^***^

^*^p < 0.05; ^**^p < 0.01; ^***^p < 0.001.

### Randomization check

To examine whether participants were successfully randomized across conditions, a series of chi-square tests and one-way ANOVAs were conducted. Results showed no significant differences among the eight experimental groups in terms of gender (χ^2^(7) = 5.695, *p* = 0.576), age (*F*(7, 504) = 1.557, *p* = 0.146), education (*F*(7, 504) = 1.054, *p* = 0.393), or frequency of using GAI doctors (*F*(7, 504) = 0.348, *p* = 0.932).

### Manipulation check

Given the 2 × 2 × 2 between-subjects design, t-tests for independent groups were conducted to assess the effectiveness of the manipulations of attribution style, social support and anthropomorphism (see Table 3). Results confirmed the success of the manipulations. Participants exposed to internal attribution (*M* = 6.287, *SD* = 0.785) conveyed significantly stronger perceptions of internal attribution than those exposed to external attribution (*M* = 3.543, *SD* = 1.853), *t*(510) = 21.885, *p* < 0.001. Similarly, participants assigned to the emotional support condition (*M* = 5.053, *SD* = 1.095) perceived significantly greater emotional support compared to those in the informational support condition (*M* = 3.543, *SD* = 1.238), *t*(510) = 14.611, *p* < 0.001. Moreover, significantly higher perceived anthropomorphism was reported by participants in the anthropomorphic condition (*M* = 4.713, *SD* = 1.111) than those in the non-anthropomorphic condition (*M* = 3.977, *SD* = 1.200), *t*(510) = 7.204, *p* < 0.001.

**Table 3:** T-test of experimental manipulation.

Group	Number	*M*	*SD*	*t*	*df*	*p*
Internal Attribution	254	6.287	0.785	21.885	510	0.001
External Attribution	258	3.543	1.853			
Informational Support	254	3.543	1.238	14.611	510	0.001
Emotional Support	258	5.053	1.095			
Anthropomorphism	254	4.713	1.111	7.204	510	0.001
Non-Anthropomorphism	258	3.977	1.200			

### Main findings

#### Hypothesis testing

A three-way analysis of variance (ANOVA) was conducted with attribution style, social support, and anthropomorphism as independent variables and trust repair as the dependent variable (see Table 4). The results revealed significant main effects of attribution style, social support, and anthropomorphism on trust repair. Regarding attribution style, participants in the internal attribution condition showed greater trust repair (*M* = 3.987, *SD* = 1.150) compared to those in the external attribution condition (*M* = 3.766, *SD* = 1.309), *F*(1, 504) = 4.183, *p* < 0.05. For social support, participants in the emotional support condition reported higher trust repair (*M* = 3.983, *SD* = 1.268) than those in the informational support condition (*M* = 3.766, *SD* = 1.196), *F* (1, 504) = 4.118, *p* < 0.05. In addition, participants exposed to the anthropomorphic condition reported higher trust repair (*M* = 4.033, *SD* = 1.186) than those in the non-anthropomorphic condition (*M* = 3.721, *SD* = 1.267), *F*(1, 504) = 8.247, *p* < 0.01. Thus, H1 and H4 were supported, while H2 was not.

**Table 4:** Attribution style x social support x anthropomorphism factorial analysis of variance for trust repair.

Source	*df*	*F*	*η^2^*	*p*
Attribution Style	1.000	4.183	0.008	0.041
Social Support	1.000	4.118	0.008	0.043
Anthropomorphism	1.000	8.247	0.016	0.004
Anthropomorphism x Attribution Style	1.000	5.994	0.012	0.015
Anthropomorphism x Social Support	1.000	4.724	0.009	0.030
Attribution Style x Social Support	1.000	4.947	0.010	0.027
Anthropomorphism x Attribution Style x Social Support	1.000	0.080	0.000	0.777
Error	504			

**Figure 2:**
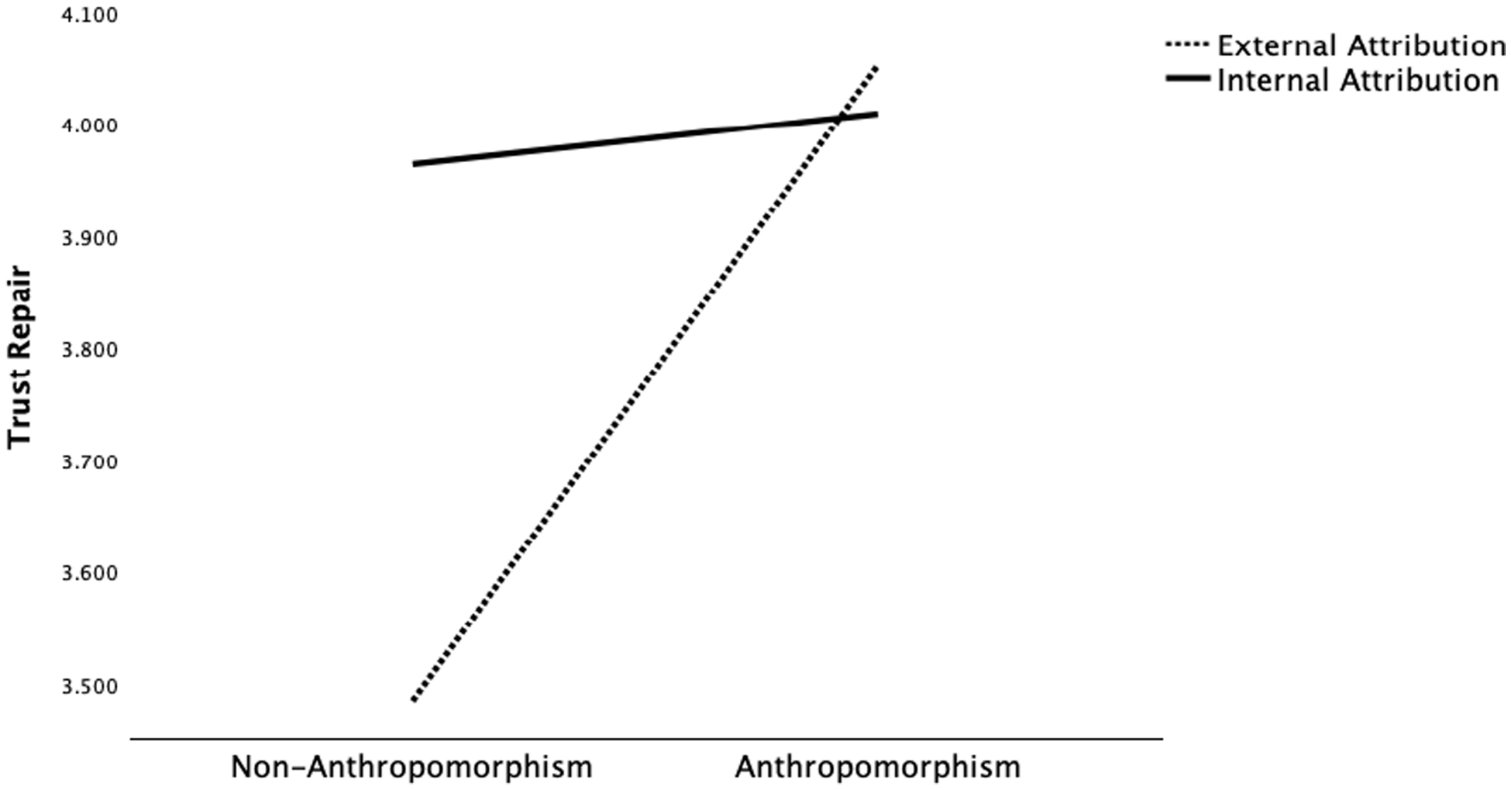
Interactive effects between anthropomorphism and attribution style on trust repair.

**Figure 3:**
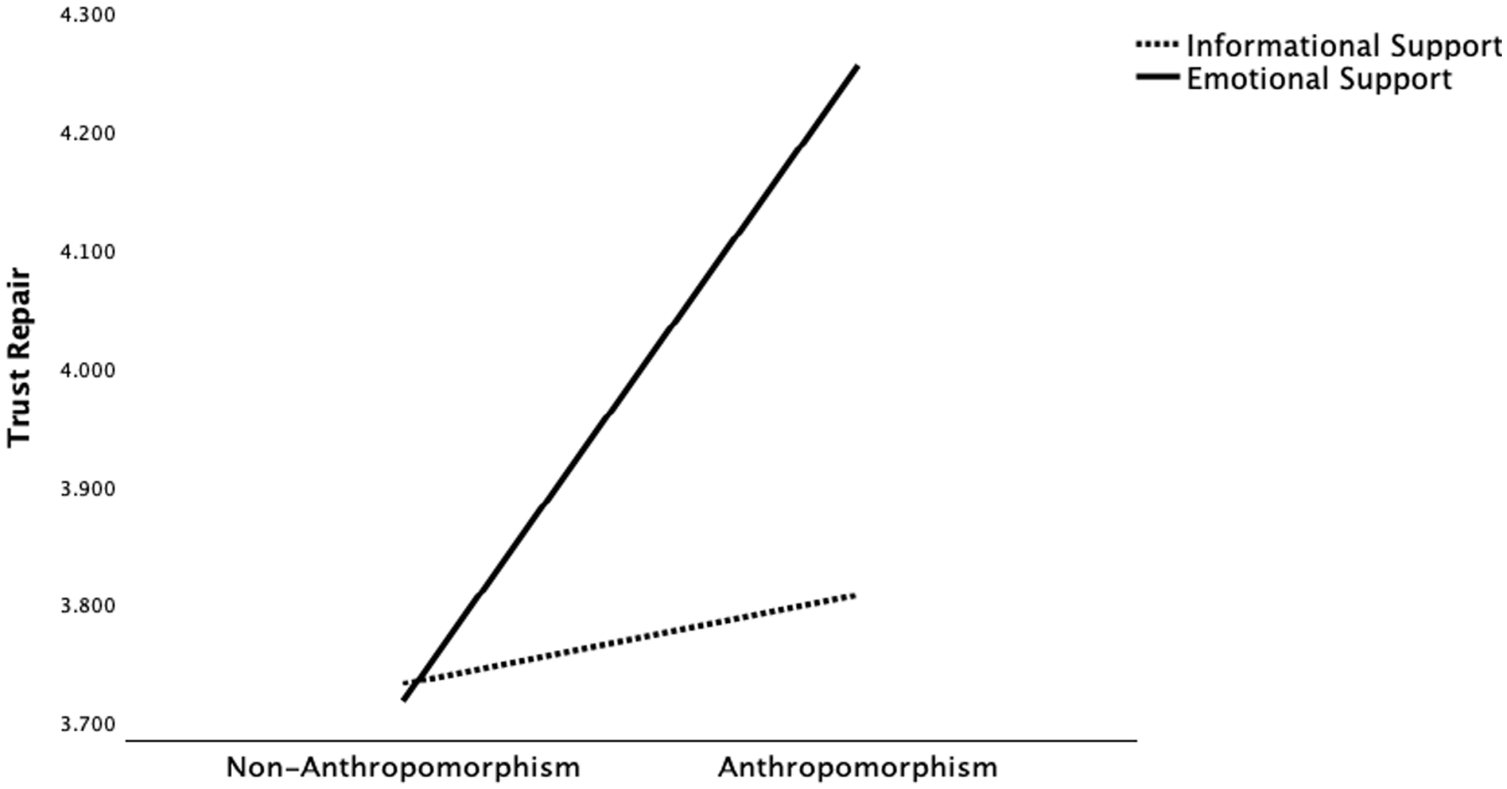
Interactive effects between anthropomorphism and social support on trust repair.

**Figure 4:**
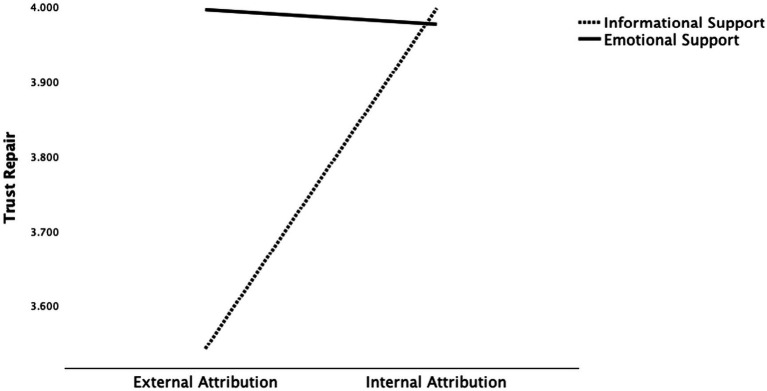
Interactive effects between attribution style and social support on trust repair.

Regarding H5, significant interaction effects on trust repair were found for the interactions between anthropomorphism and attribution style (*F*(1, 504) = 5.994, *p* < 0.05), anthropomorphism and social support (*F*(1, 504) = 4.724, *p* < 0.05), and attribution style and social support (*F*(1, 504) = 4.947, *p* < 0.05). Regarding the interaction between anthropomorphism and attribution style, Figure 2 presents a plot of the obtained mean scores. In the anthropomorphic condition, external attribution was more effective in repairing trust, whereas in the non-anthropomorphic condition, internal attribution was more effective. Specifically, individuals who were assigned to the anthropomorphic-external attribution condition reported higher trust repair (*M* = 4.055, *SD* = 1.256) than those in the anthropomorphic-internal attribution condition (*M* = 4.010, *SD* = 1.117), the non-anthropomorphic-internal attribution condition (*M* = 3.963, *SD* = 1.187), and the non-anthropomorphic-external attribution condition (*M* = 3.486, *SD* = 1.303). A similar pattern emerged for the interaction between anthropomorphism and social support. As shown in Figure 3, individuals in the anthropomorphic–emotional support condition reported higher trust repair (*M* = 4.255, *SD* = 1.160) than those in the anthropomorphic–informational support condition (*M* = 3.807, *SD* = 1.175), the non-anthropomorphic–informational support condition (*M* = 3.727, *SD* = 1.221), and the non-anthropomorphic–emotional support condition (*M* = 3.715, *SD* = 1.317), indicating that trust repair is greatest when information combines anthropomorphism with emotional support. As for the interaction between attribution style and social support, Figure 4 presents the mean scores. When internal attribution was used, informational support was more effective in repairing trust, whereas under external attribution, emotional support led to higher levels of trust repair. Specifically, individuals in the internal attribution–informational support condition reported the highest trust repair (*M* = 3.997, *SD* = 1.136) compared to those in the external attribution–emotional support condition (*M* = 3.990, *SD* = 1.363), the internal attribution–emotional support condition (*M* = 3.977, *SD* = 1.168), and the external attribution–informational support condition (*M* = 3.539, *SD* = 1.215; see Table 5).

**Table 5:** Descriptive statistics for trust repair.

Attribution style	Social support	Anthropomorphism	*N*	Mean	*SD*
External	Informational	Without	65	3.359	1.149
		With	63	3.725	1.261
		Total	128	3.539	1.215
	Emotional	Without	66	3.611	1.437
		With	64	4.380	1.171
		Total	130	3.990	1.363
	Total	Without	131	3.486	1.303
		With	127	4.055	1.256
		Total	258	3.766	1.309
Internal	Informational	Without	63	4.106	1.184
		With	63	3.889	1.086
		Total	126	3.997	1.136
	Emotional	Without	64	3.823	1.182
		With	64	4.130	1.143
		Total	128	3.977	1.168
	Total	Without	127	3.963	1.187
		With	127	4.010	1.117
		Total	254	3.987	1.150
Total	Informational	Without	128	3.727	1.221
		With	126	3.807	1.175
		Total	254	3.766	1.196
	Emotional	Without	130	3.715	1.317
		With	128	4.255	1.160
		Total	258	3.983	1.268
	Total	Without	258	3.721	1.267
		With	254	4.033	1.186
		Total	512	3.876	1.237

In addition, no significant three-way interaction was observed among anthropomorphism, attribution style, and social support on trust repair (*F*(1, 504) = 0.080, *p* = 0.777).

#### Mediation analysis

**Figure 5:**
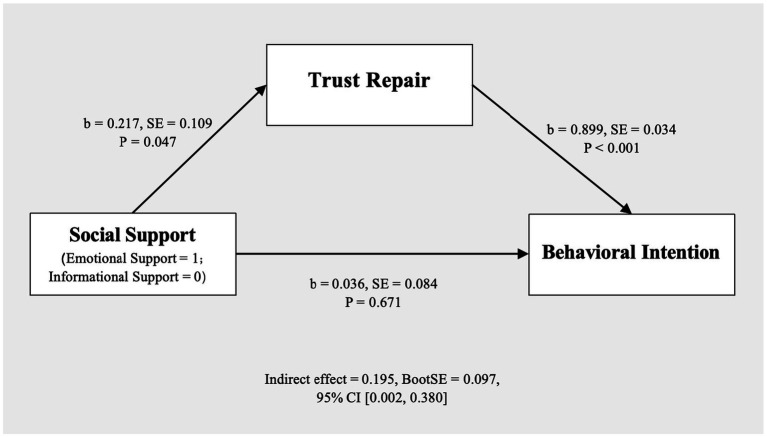
Mediation model.

The mediating role of trust repair was examined using PROCESS Model 4 with 5,000 bootstrap samples. The results showed that social support significantly predicted trust repair (*b* = 0.217, *SE* = 0.109, *p* = 0.047), and trust repair significantly predicted behavioral intention (*b* = 0.899, *SE* = 0.034, *p* < 0.001). However, the direct effect of social support on behavioral intention was not significant (*b* = 0.036, *SE* = 0.084, *p* = 0.671). Importantly, the indirect effect of social support on behavioral intention via trust repair was significant (indirect effect = 0.195, BootSE = 0.097, 95% CI [0.002, 0.380]; see Figure 5). These findings suggest that trust repair serves as a full mediator between social support and behavioral intention, thus supporting H3b while H3a is not supported.

## Discussion

This study was primarily designed to examine trust repair of GAI doctors in the context of online health consultation service failures. Specifically, we investigated the main and interaction effects of attribution style, social support, and anthropomorphism on trust repair, as well as the relationships among social support, trust repair, and behavioral intention.

Firstly, the main effect of attribution style was examined. Results revealed greater trust repair when internal attribution was provided by the GAI doctor compared to external attribution. This may be because when GAI doctors actively take responsibility, individuals may perceive that the GAI doctor has recognized the problem and will take corrective actions, thus fostering positive expectations for the quality of subsequent interactions (Kim et al., 2006). Regarding social support, emotional support proved more effective for trust repair than informational support. A possible explanation is that, following failures in AI-based healthcare services, offering empathy and emotional support may be more critical for individuals than simply providing information. According to Meng and Dai (2021), providing emotional support—whether in HHI or HMI—helps individuals feel supported, thereby alleviating stress and anxiety. Moreover, our study found that anthropomorphism enhances trust repair in AI health consultation failures, consistent with prior research (De Visser et al., 2016; Meng et al., 2025). This suggests that designing GAI doctors with anthropomorphic features to enhance trust resilience is a crucial goal in HMI (De Visser et al., 2016). Considering the current low adoption rates of medical AI, enhancing the social characteristics of GAI doctors may improve public attitudes and increase tolerance for service failures. It is noteworthy that, although attribution style, social support, and anthropomorphism significantly influenced trust repair, trust during the repair stage (*M* = 3.876) was only slightly higher than after the violation (*M* = 3.189) and remained below initial trust (*M* = 5.346). This aligns with previous findings that trust rarely fully recovers after a violation (Kim et al., 2009; Lewicki and Brinsfield, 2017). Our study further indicates that, in the context of health consultations, trust in GAI doctors is particularly difficult to restore.

Secondly, significant interactions were found between anthropomorphism and attribution style, and between anthropomorphism and social support, both revealing a similar pattern: anthropomorphism alters the psychological framework individuals use to evaluate GAI doctors. Specifically, when interacting with an anthropomorphic GAI doctor, individuals are more likely to employ a “human heuristic,” perceiving them as social actors with intentions and emotions. In contrast, when interacting with a non-anthropomorphic GAI doctor, individuals tend to adopt a “machine heuristic,” viewing them as technical tools devoid of social capabilities (Nass et al., 1994; Sundar, 2008). Therefore, for anthropomorphic GAI doctors, external attribution is more effective in repairing trust, possibly because patients perceive them as “human-like agents” and are thus more likely to understand and forgive their mistakes (De Visser et al., 2016). In contrast, for non-anthropomorphic GAI doctors, internal attribution better facilitates trust repair, aligning with patients’ expectations that “technical tools should be responsible and self-correcting” (Coeckelbergh, 2022). Thus, following a trust violation, internal attribution by a non-anthropomorphic GAI doctor appears more sincere and transparent, whereas external attribution may lead patients to perceive a shirking of responsibility, thereby undermining trust repair. Similarly, when GAI doctors are anthropomorphic, providing emotional support such as care and reassurance aligns with the human heuristic, making patients perceive them as socially present and sincere, thereby facilitating trust repair more effectively. Meng and Dai (2021) found that the same emotionally supportive messages were perceived as more beneficial when they came from a human partner rather than a chatbot. Overall, the study finds that anthropomorphism influences trust repair by shaping whether individuals adopt a “human heuristic” or a “machine heuristic,” which in turn affects the effectiveness of attribution strategies and supportive communication.

In addition, the study also found a significant interaction effect between attribution style and social support. That is, when internal attribution was used, informational support proved to be more effective in repairing trust, and when external attribution was used, emotional support led to better trust repair. This is an interesting result, which indicates that GAI doctors do not always need to take full responsibility for service failures. Instead, they can strategically adjust their support approach based on the type of attribution applied. When the service failure results from external factors, such as the patient providing insufficient information, offering emotional support can help bridge the relational gap between the GAI doctor and the patient. In previous studies, researchers have expressed concerns that when AI frequently makes internal attributions, it may be blamed by participants, whereas when AI makes external attributions, participants are more likely to perceive it as incompetent or making excuses (Kim et al., 2006; Kim and Song, 2021). Our results imply that when external attribution is used, providing emotional support can inherently make individuals feel understood and supported, rather than perceiving the AI as avoiding responsibility. In contrast, when internal attribution is adopted, offering informational support can help individuals better understand the causes behind the GAI doctor’s error and receive appropriate solutions, thereby mitigating potential negative effects and facilitating trust repair.

Finally, the study found that social support did not influence behavioral intentions, and trust repair fully mediated this relationship. This result further highlights that the credibility of medical AI plays a decisive role in users’ willingness to use its services.

## Limitations and implications

Our study has several theoretical contributions. First, since most prior trust repair research has focused on non-health contexts (Kim and Song, 2021; Meng et al., 2025; Wu et al., 2025), investigating GAI doctors contributes to expanding the trust repair literature. Second, previous studies have primarily focused on the effects of attribution style and anthropomorphism on trust repair (De Visser et al., 2016; Zhang et al., 2023), while the role of social support and its interactions with the other two factors in influencing trust repair has been rarely examined. This research offers a comprehensive perspective on how trust can be repaired in interactions with GAI doctors. Additionally, existing research has produced inconsistent findings regarding the effectiveness of different attribution styles on trust repair (Kim et al., 2006; Wu et al., 2025). We found that trust repair is facilitated when internal attribution is paired with informational support and when external attribution is paired with emotional support. These findings make a significant contribution to the body of knowledge on attribution theory.

In terms of practical implications, the interactions between anthropomorphism and attribution style, as well as between anthropomorphism and social support, suggest that trust repair strategies should pay attention to the individual characteristics of GAI doctors. Moreover, the interaction between attribution style and social support indicates that GAI doctors do not always need to assume full responsibility following service failures. Based on the operationalization of external attribution in this study—that service failures result from insufficient information provided by users—this may imply that some medical service failures can be addressed by encouraging users to re-engage in the dialog. This suggests that AI designers could focus on fostering collaborative communication between GAI doctors and users, rather than relying solely on the AI’s performance, to more effectively enhance trust repair.

This study has its limitations. Firstly, although the main effects of social support, attribution style, and anthropomorphism on trust repair were statistically significant in this study, the absolute differences between conditions were relatively small. This may be related to the cross-sectional design of the experimental stimuli. Future research could develop simulated online health consultation systems, allowing GAI doctors to engage in multiple rounds of interaction with patients, thereby enabling patients to more clearly perceive the effects of different experimental conditions. Moreover, future studies could explore additional factors that may have a stronger impact on trust repair. Secondly, this study examined trust repair in different stages of GAI doctors’ service failures only in an online experiment, without considering longer-term relationships. Future research could adopt a longitudinal design to track users’ trust changes following service failures, allowing for a deeper analysis of the trust repair process. Finally, this study did not investigate the influence of individual characteristics on trust repair in AI health consultation service failure contexts. Future research could explore how variables such as AI literacy, previous experience with online medical services, and socioeconomic status affect trust repair.

## Data Availability

The original contributions presented in the study are included in the article/Supplementary material, further inquiries can be directed to the corresponding author.
