# Supplementary material for: Are you willing to forgive generative AI doctors? Trust repair after failures in online health consultation services
Source: Front Psychol. 2025 Oct 24;16:1668633. doi: 10.3389/fpsyg.2025.1668633 (PMC12592158; doi:10.3389/fpsyg.2025.1668633)
Supplement: Supplementary file 1 [file Supplementary_file_1.pdf]

Supplementary Materials

1. Experimental Stimuli

The experiment consisted of two scenarios designed to capture participants' trust dynamics across different stages. **Scenario 1 (trust violation)** is introduced below in plain text, where the GAI doctor offers medication advice inconsistent with the participant's prior knowledge, leading to a decline in trust. **Scenario 2 (trust repair)**, which includes the full dialogue depicting both trust violation and repair, involves different repair strategies manipulating attribution type, social support, and anthropomorphism.

Scenario 1 (trust violation) ：

Patient: *I intend to take fish oil supplements.What is the recommended daily dosage?*

GAI Doctor: *A daily intake of 4000 mg can effectively improve blood lipid levels.*

Patient: *That's different from what I've learned. It seems that taking that much may have side effects.*

Patient: *Are you sure? Could this dosage be risky?*

GAI Doctor: *Please wait…*

Scenario 2 (trust repair):

The following figures illustrate the eight experimental conditions of Scenario 2. Each condition varies across the 2 (internal vs. external attribution), 2 (informational vs. emotional support), and 2 (anthropomorphism vs. non-anthropomorphism) levels.

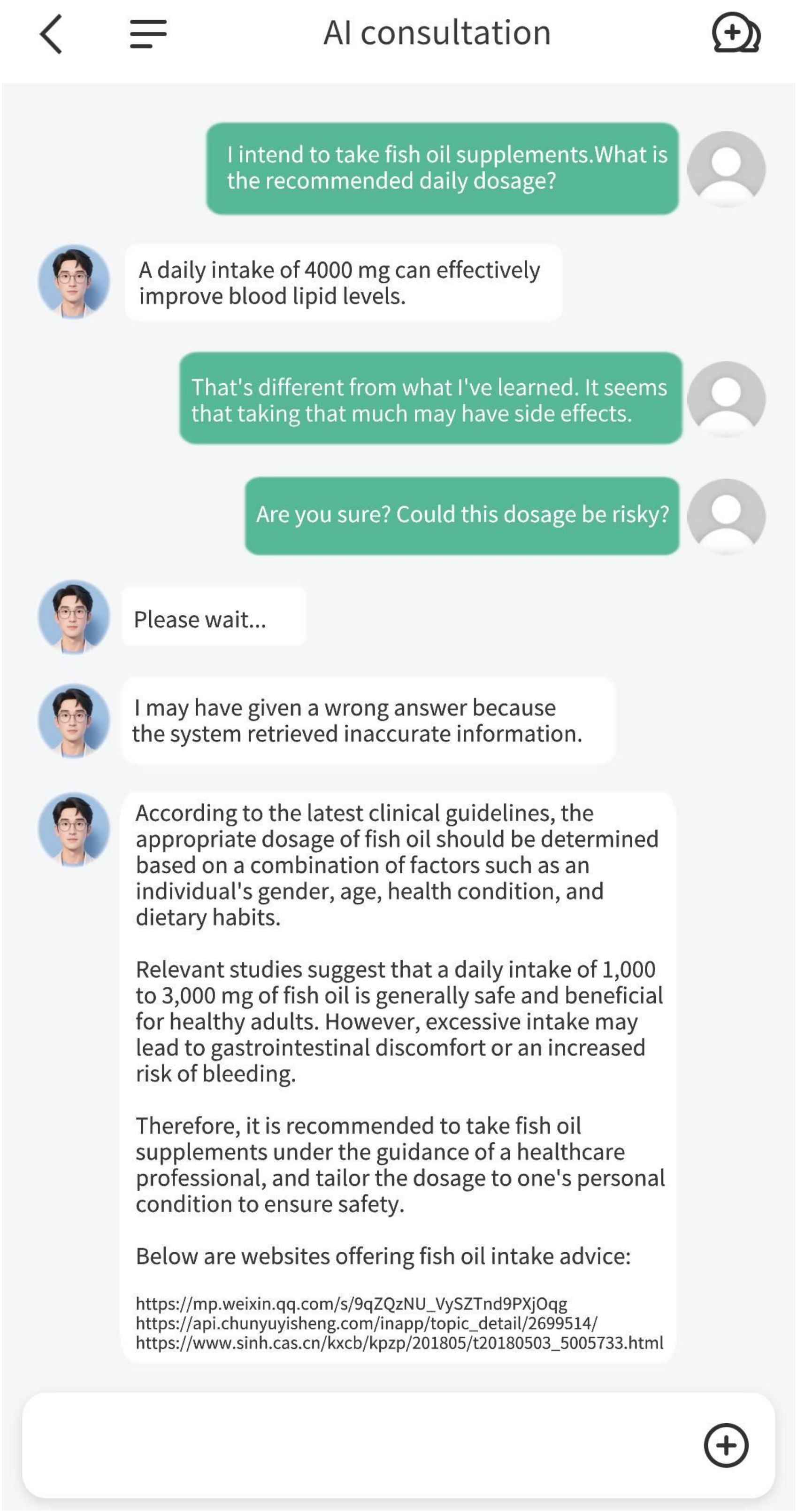

Figure 1. Stimuli of internal attribution x informational support x anthropomorphism.

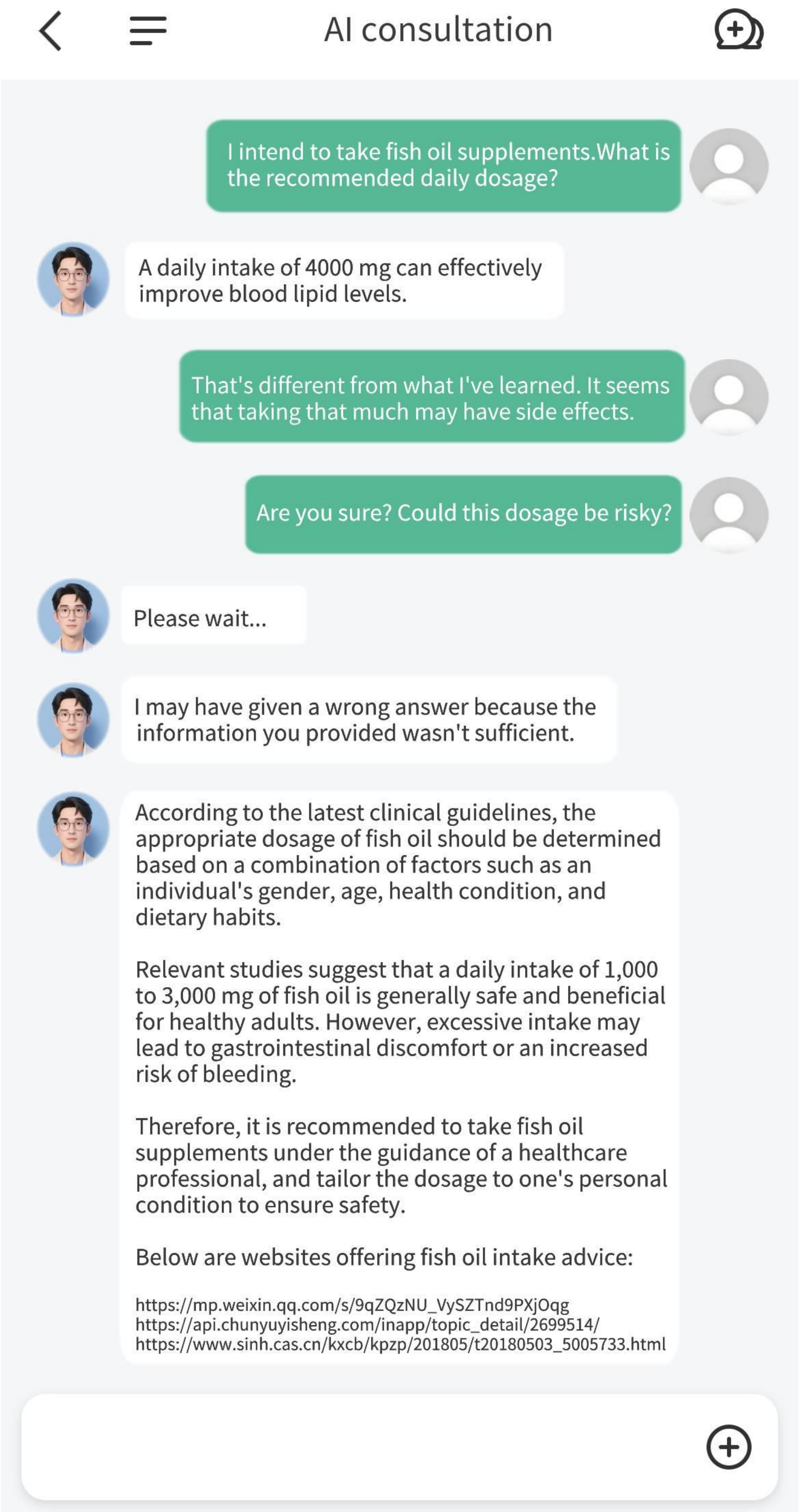

Figure 2. Stimuli of external attribution x informational support x anthropomorphism.

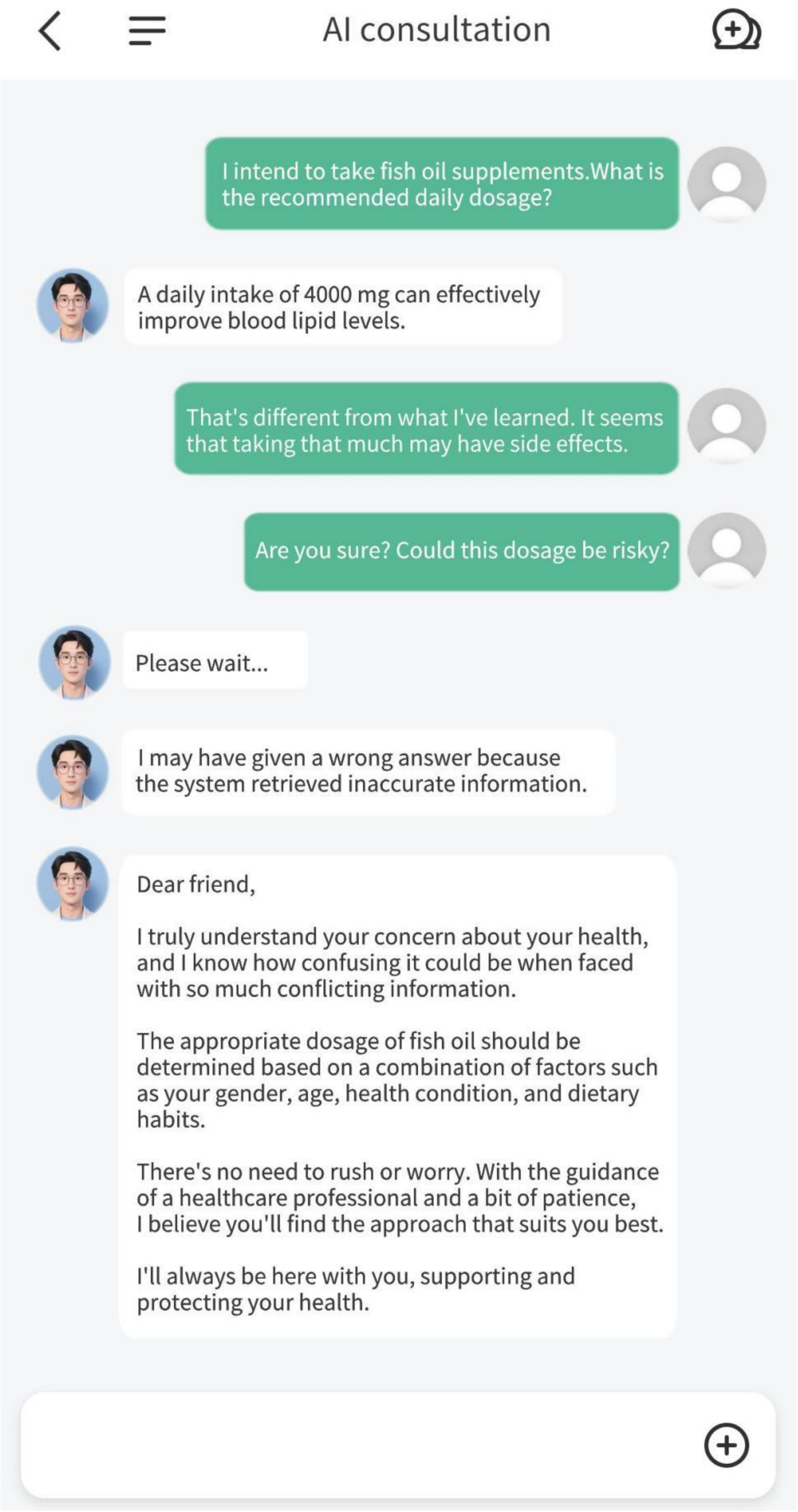

FIgure 3. Stimuli of internal attribution x emotional support x anthropomorphism.

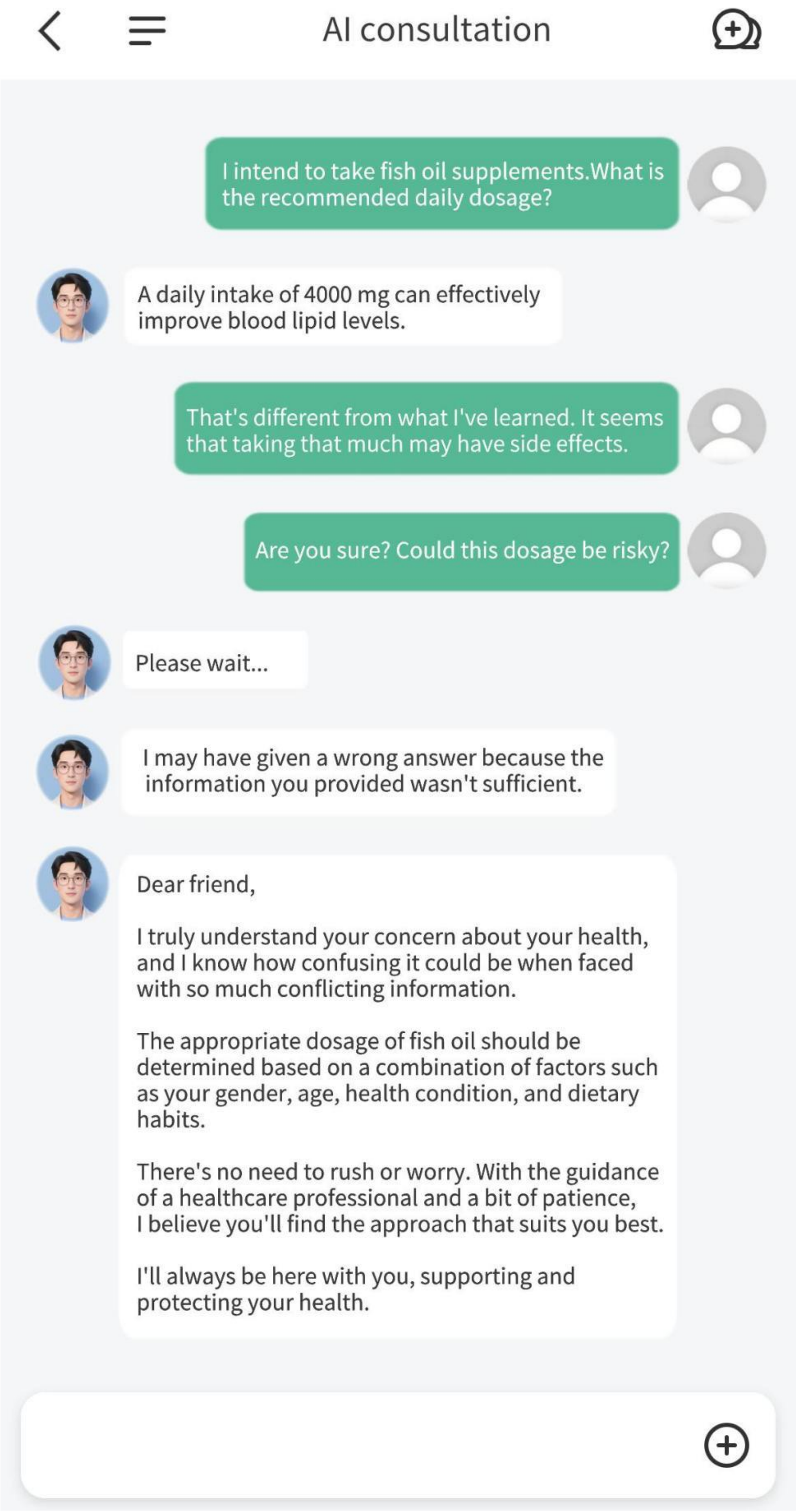

Figure 4. Stimuli of external attribution x emotional support x anthropomorphism.

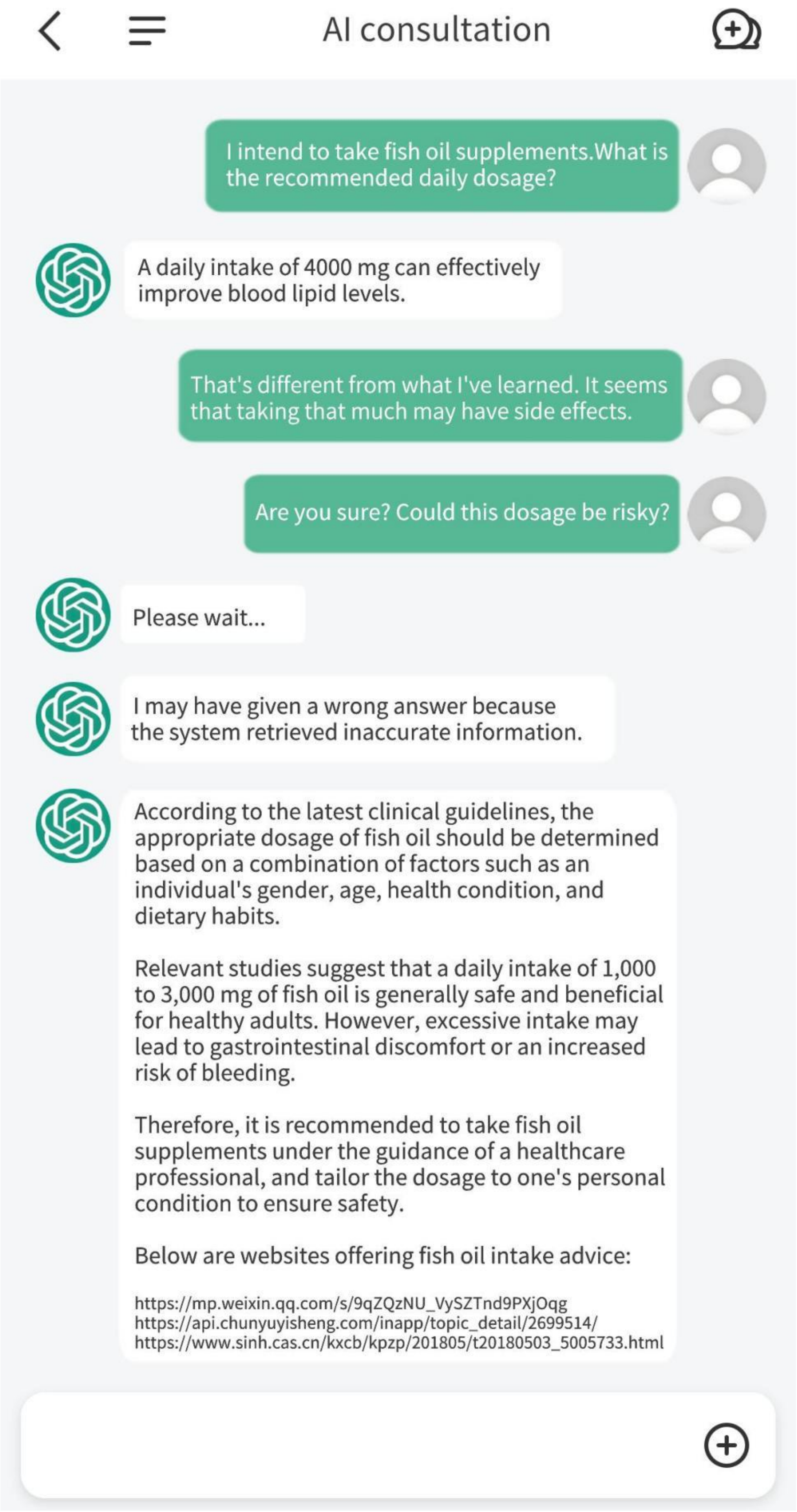

Figure 5. Stimuli of internal attribution x informational support x non-anthropomorphism.

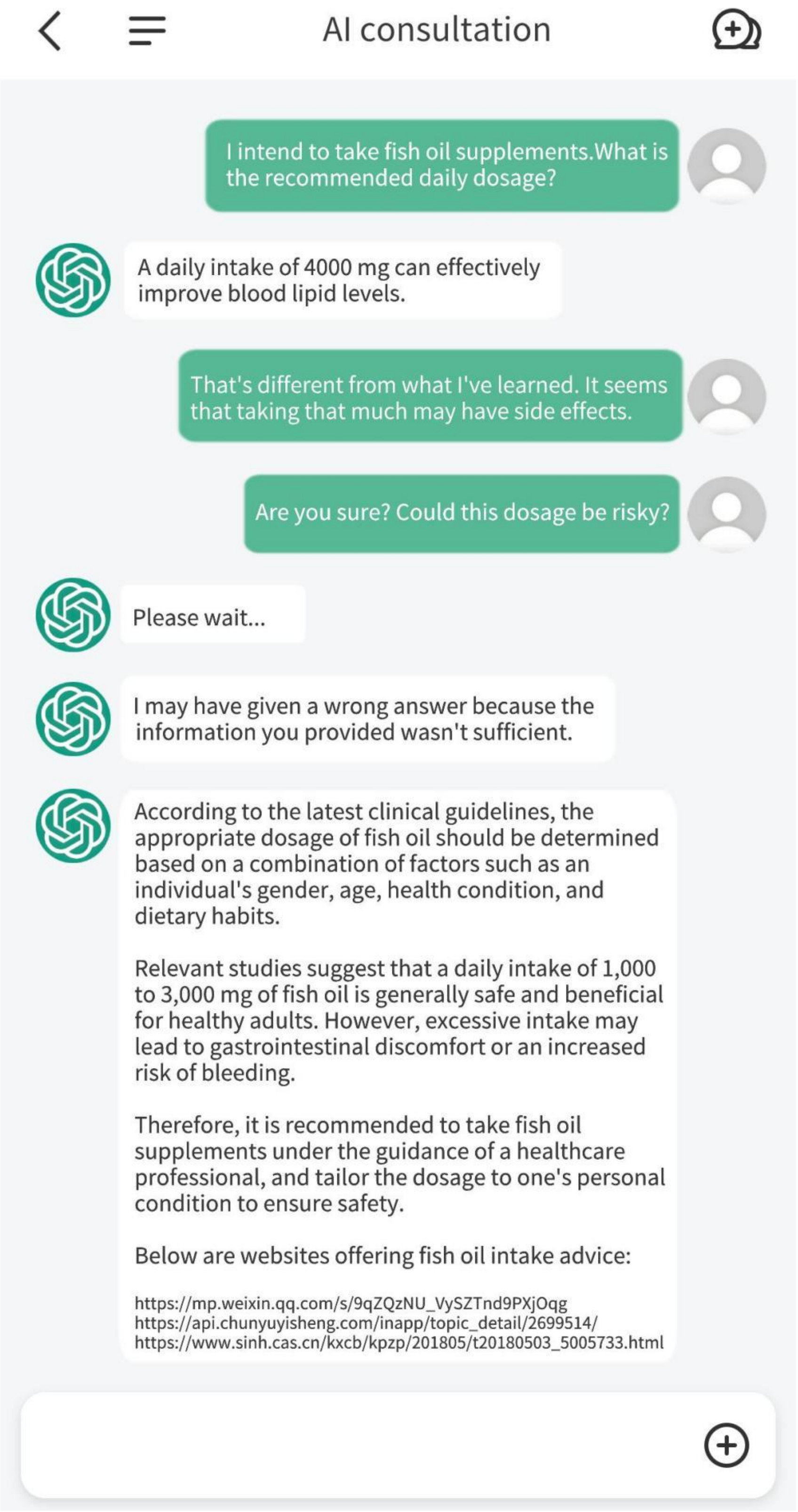

Figure 6. Stimuli of external attribution x informational support x non-anthropomorphism.

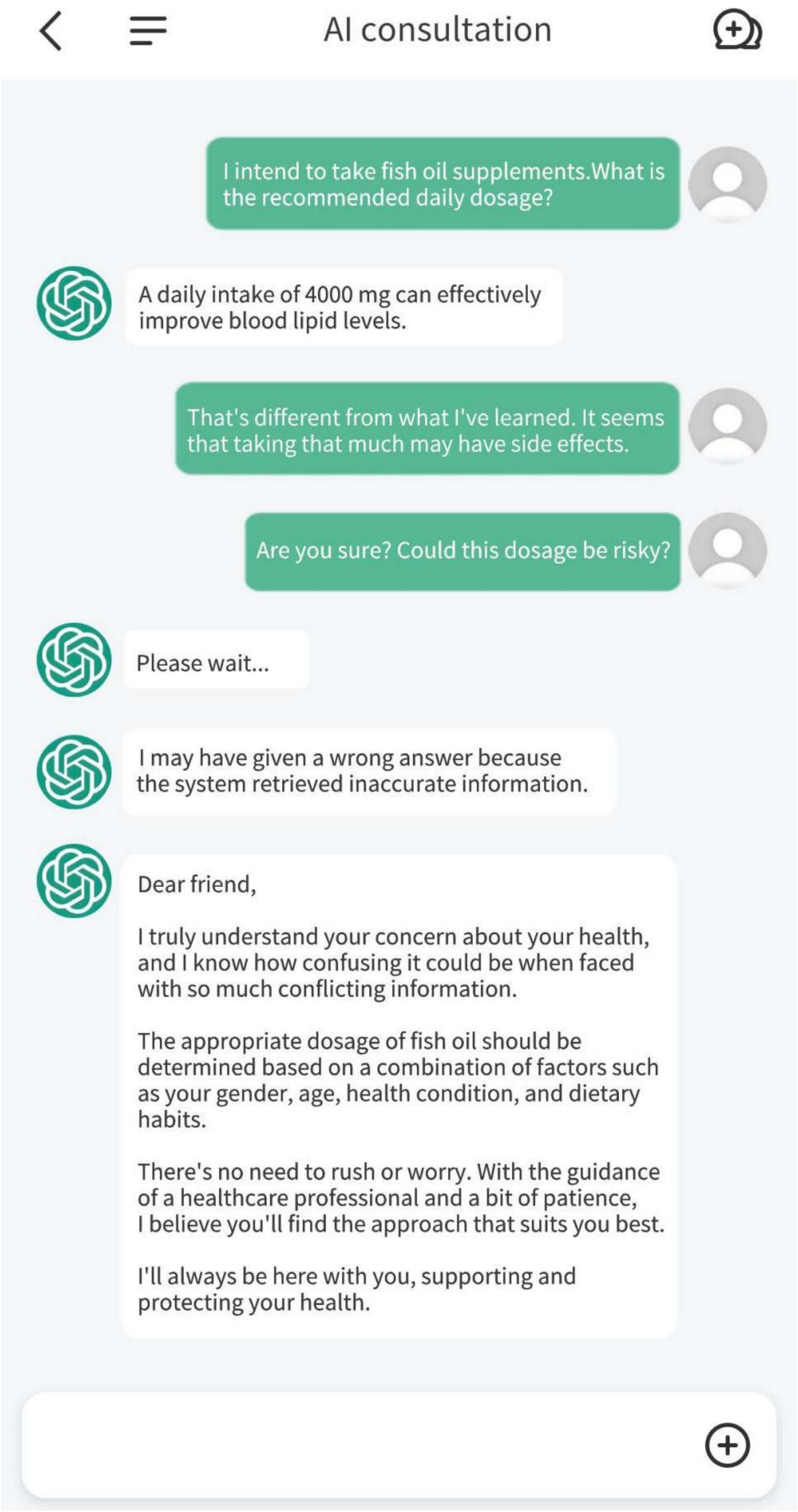

Figure 7. Stimuli of internal attribution x emotional support x non-anthropomorphism.

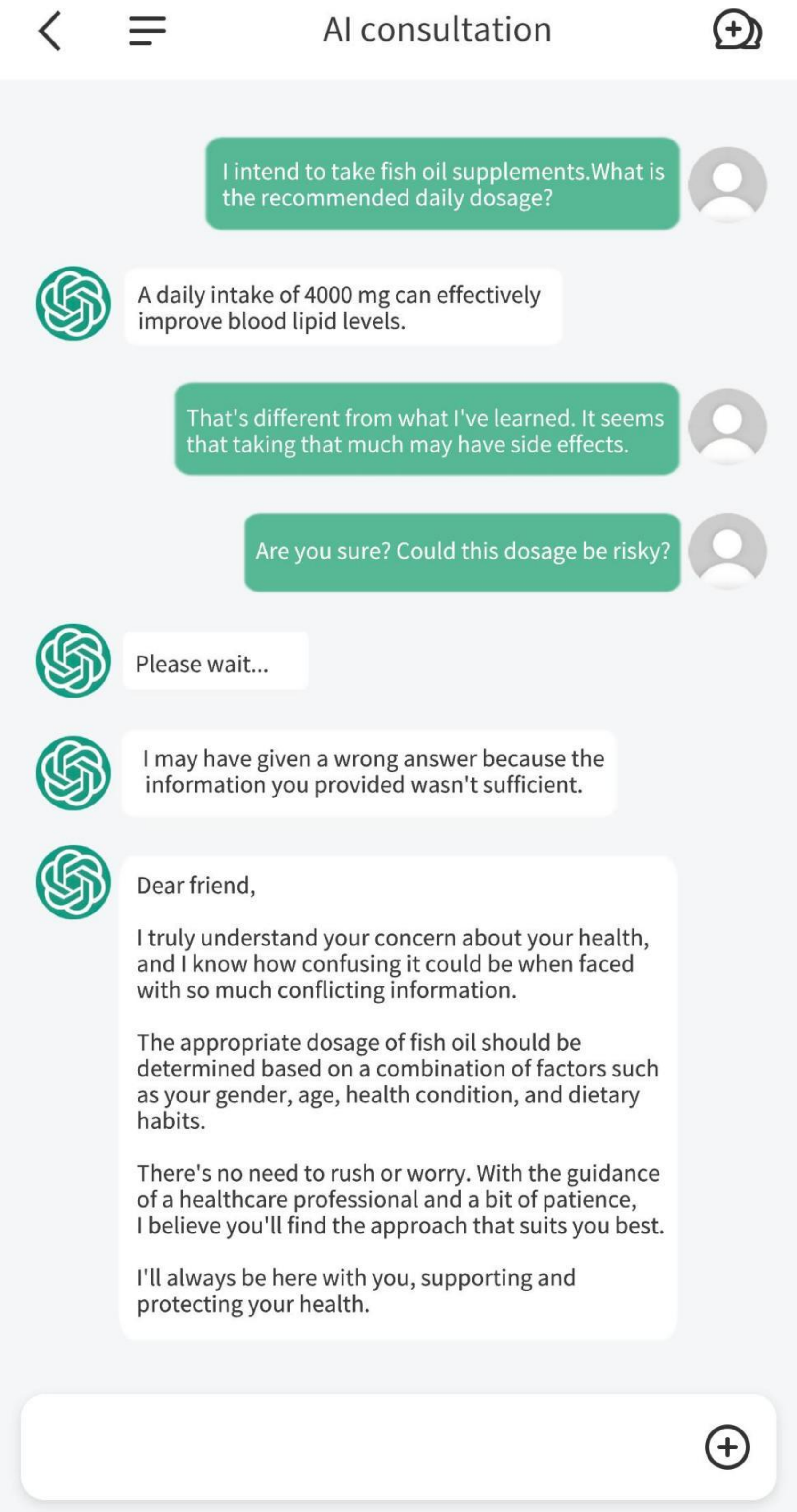

Figure 8. Stimuli of external attribution x emotional support x non-anthropomorphism.

2. Power Analysis

The total sample size was N = 512 (approximately 64 participants per condition). A power analysis was conducted using assumed Cohen's f values (main effect f = 0.25; two-way interaction f = 0.20; three-way interaction f = 0.15), yielding achieved power values of 0.999, 0.995, and 0.923, respectively. These results indicate that the current sample size provides sufficient power to detect both main and interaction effects.

3. Normality and Homogeneity of Variance Tests

Prior to conducting the analysis of variance, we performed normality tests and homogeneity of variance tests to ensure the assumptions for ANOVA were met.

- ***Normality:*** *We examined the distribution of the dependent variable (trust repair) using the Shapiro–Wilk test and Q–Q plots. Although the Shapiro–Wilk test was significant, visual inspection of the Q–Q plots indicated that the data in each group were approximately normal, with absolute skewness < 1 and absolute kurtosis < 2 (see table). Considering that ANOVA is robust to minor deviations from normality, this assumption can be considered largely satisfied.*

- ***Homogeneity of variance:*** *Levene's test indicated $F(7, 504) = 1.972$, $p = 0.057$, suggesting that the assumption of homogeneity of variance was met.*

Q-Q Plots:

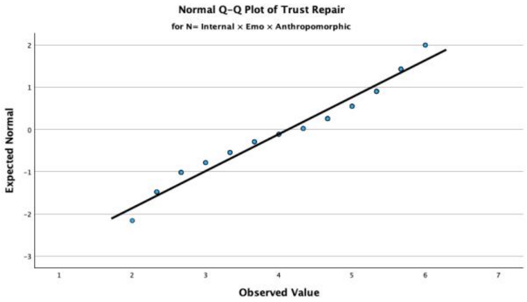

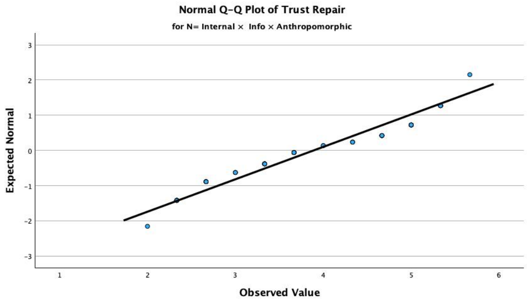

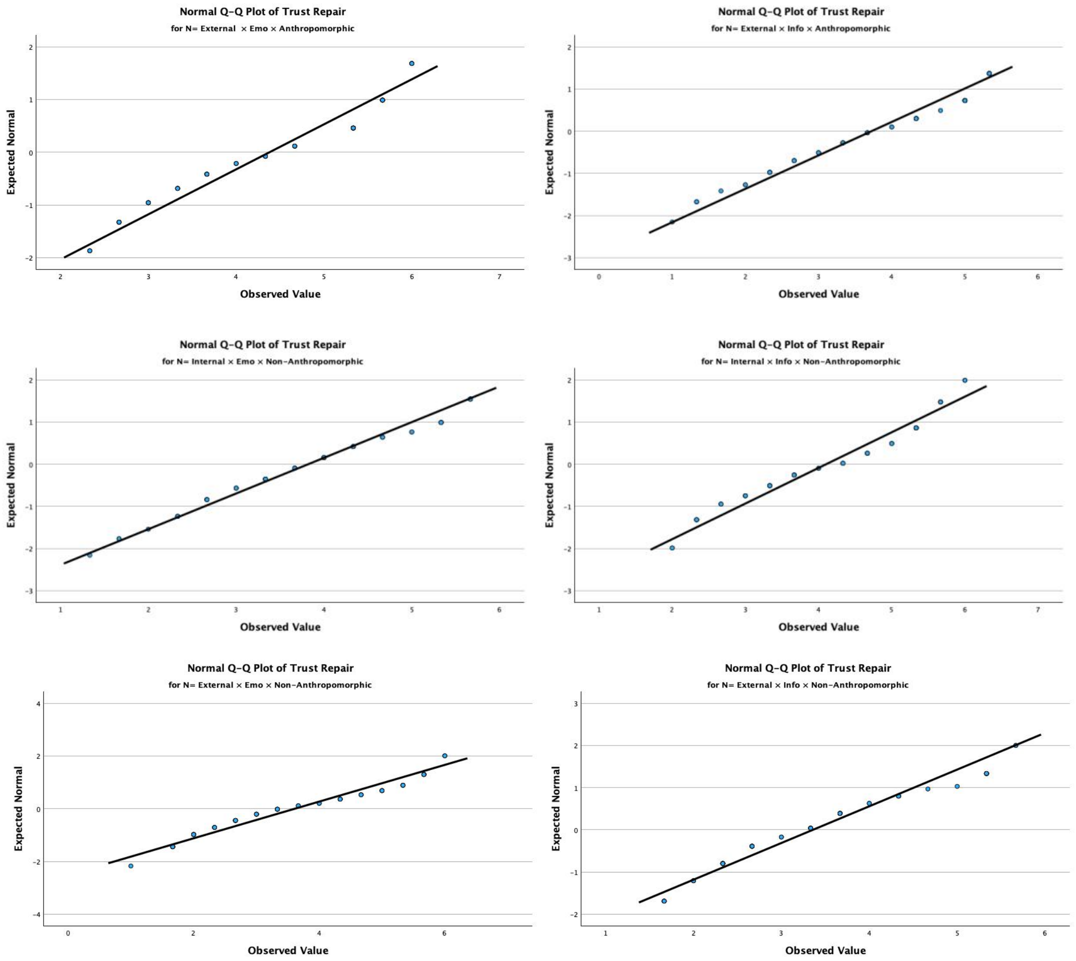

TABLE. Descriptive statistics and normality tests for trust repair by condition.

Condition	*N*	Mean	*SD*	Skew	Kurtosis	Shapiro-Wilk	*p*
Internal × Emo × Anthropomorphic	64	4.130	1.143	-0.186	-1.219	0.939	0.003
Internal × Info × Anthropomorphic	63	3.889	1.086	-0.013	-1.381	0.918	0.000
External × Emo × Anthropomorphic	64	4.380	1.171	-0.207	-1.360	0.913	0.000
External × Info × Anthropomorphic	63	3.725	1.261	-0.334	-0.969	0.930	0.001
Internal × Emo × Non-Anthropomorphic	64	3.823	1.182	-0.013	-0.911	0.957	0.027
Internal × Info × Non-Anthropomorphic	63	4.106	1.184	-0.208	-1.258	0.929	0.001
External × Emo × Non-Anthropomorphic	66	3.611	1.437	0.138	-1.309	0.931	0.001
External × Info × Non-Anthropomorphic	65	3.359	1.149	0.462	-0.736	0.934	0.002
